# Accumulation of Wildtype and ALS-Linked Mutated VAPB Impairs Activity of the Proteasome

**DOI:** 10.1371/journal.pone.0026066

**Published:** 2011-10-05

**Authors:** Anice Moumen, Isabelle Virard, Cédric Raoul

**Affiliations:** 1 Inserm-Avenir team, the Mediterranean Institute of Neurobiology, INMED, Marseille, France; 2 Université de la Méditerranée, UMR S901 Aix-Marseille 2, Marseille, France; University of New South Wales, Australia

## Abstract

Cellular homeostasis relies on a tight control of protein synthesis, folding and degradation, in which the endoplasmic reticulum (ER) quality control and the ubiquitin proteasome system (UPS) have an instrumental function. ER stress and aberrant accumulation of misfolded proteins represent a pathological signature of amyotrophic lateral sclerosis (ALS), a fatal paralytic disorder caused by the selective degeneration of motoneurons in the brain and spinal cord. Mutations in the ER-resident protein VAPB have been associated with familial forms of the disease. ALS-linked mutations cause VAPB to form cytoplasmic aggregates. We previously demonstrated that viral-mediated expression of both wildtype and mutant human VAPB (hVAPB) leads to an ER stress response that contributes to the selective death of motoneurons. However, the mechanisms behind ER stress, defective UPS and hVAPB-associated motoneuron degeneration remain elusive. Here, we show that the overexpression of wildtype and mutated hVAPB, which is found to be less stable than the wildtype protein, leads to the abnormal accumulation of ubiquitin and ubiquitin-like protein conjugates in non-human primate cells. We observed that overexpression of both forms of hVAPB elicited an ER stress response. Treatment of wildtype and mutated hVAPB expressing cells with the ER stress inhibitor salubrinal diminished the burden of ubiquitinated proteins, suggesting that ER stress contributes to the impairment of proteasome function. We also found that both wildtype and mutated hVAPB can associate with the 20S proteasome, which was found to accumulate at the ER with wildtype hVAPB or in mutant hVAPB aggregates. Our results suggest that ER stress and corruption of the proteasome function might contribute to the aberrant protein homeostasis associated with hVAPB.

## Introduction

Amyotrophic lateral sclerosis (ALS) is an adult-onset neurodegenerative disease, which primarily affects motoneurons in the cortex, brainstem and spinal cord. Symptoms begin with a focal muscle weakness and wasting, which irrevocably spreads to complete paralysis and leads to death within 3 to 5 years. ALS occurs either in a predominant sporadic form, or less frequently, in an inherited, familial form, both being clinically indistinguishable. Like in many other neurodegenerative disorders, neuronal cytoplasmic proteinaceous aggregates are a pathological signature of the disease. These protein deposits, referred to as Bunina bodies, Lewy body-like or hyaline inclusions are suggested to play a decisive role in the pathogenesis of both sporadic and familial ALS [1], [2].

In healthy cells, protein quality control systems in the cytoplasm and endoplasmic reticulum (ER) ensure a tight regulation of protein concentration and folding through selective clearance mechanisms. In particular, the proteasome, a large multicatalytic complex, plays an instrumental role in eliminating improperly folded or damaged proteins. Proteins targeted for destruction are covalently marked at lysine residues by ubiquitin, a 76 amino-acid polypeptide, through multi-enzymatic sequential transfer in order to be recognized by the proteasomal degradative machinery [3]. An impairment of the ubiquitin-proteasome system (UPS) has been proposed to lead to the accumulation of ubiquitin-conjugated proteins and the formation of aggregates [4], [5]. In both familial and sporadic ALS as well as in a proportion of ALS with frontotemporal dementia (FTD), inclusions immunoreactive for ubiquitin are observed in motoneurons [6], [7], [8], [9]. However, the mechanisms by which ALS-causing factors compromise protein homeostasis and lead to intracellular aggregates remain elusive.

Mutations in the vesicle-associated membrane protein (VAMP)-associated protein B (VAPB) have been associated with ALS [10]. VAPB is a type II integral membrane protein that mainly locates at the endoplasmic reticulum (ER). VAPB has been proposed to act in the regulation of COPI-mediated protein transport within the Golgi apparatus and from the Golgi back to the ER [11]. VAPB has been documented to maintain the structural and functional integrity of the Golgi through the control of lipid transport [12], and of the ER probably through its bridging to the microtubule network [13]. Another VAPB function relates to the modulation of the unfolded protein response (UPR), though the precise role of VAPB in the control of the UPR remains unclear [14], [15], [16], [17].

The two ALS-associated missense mutations (P56S and T46I) in VAPB that have been identified so far lead to the formation of dense and insoluble cytosolic VAPB aggregates [10], [18]. The presence of mutant VAPB aggregates is accompanied by the formation of aberrant ER structures [19], [20], [21] and an ineffectual UPR [14], [15], [16], [17], [18]. We demonstrated that the overexpression of both wildtype and mutated VAPB disturbs Ca^2+^ homeostasis in motoneurons and that this contributes to their selective degeneration [17]. Ubiquitinated aggregates are also found in cells expressing VAPB mutants, though these aggregates do not or only rarely colocalize with VAPB aggregates in mammalian cells [18], [21], as well as in spinal motoneurons of mutated VAPB transgenic mice [22]. This observation suggests that a more global alteration of the protein clearance system could participate in the pathogenic mechanisms of VAPB.

Here, we show that the overexpression of both wildtype and mutated hVAPB leads to the accumulation of proteins targeted for proteasomal degradation. We provide evidence that the ER stress response elicited by the forced expression of hVAPB^WT^ and hVAPB^P56S^ partially compromises the protein clearance machinery. In addition, we found that both forms of hVAPB interact with the proteasome suggesting that another inhibitory mechanism of proteasomal activity could include the trapping of the proteasome. We propose that disturbance of the UPS activity that might occur through ER stress and sequestration of the proteasome contributes to the pathogenic process in VAPB-associated motoneuron disease.

## Results

### hVAPB localizes at the ER and with components of the secretory pathway

**Figure 1:**
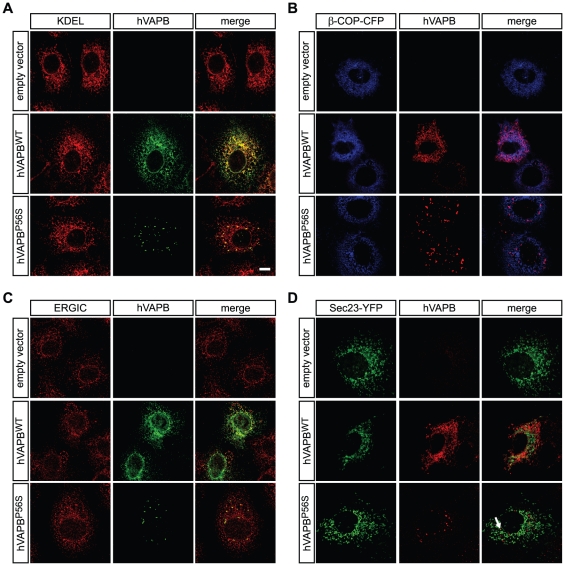
Wildtype and mutated hVAPB associate with components of the secretory pathway in non-human primate cells. (A–D) Thirty-six hours following transfection of COS-7 cells, hVAPB^WT^ and hVAPB^P56S^ mainly colocalize with components of the secretory pathway as demonstrated by the immunostaining of hVAPB with the ER marker KDEL (A), the COPI vesicle marker β-COP-CFP (B) and ERGIC marker ERGIC-53 (C). hVAPB^P56S^ forms cytoplasmic aggregates that colocalize with ER, COPI and ERGIC markers. Both hVAPB^WT^ and hVAPB^P56S^ seldom colocalize (white arrow) with the COPII marker Sec23-YFP (D). Scale bar, 20 µm.

To better understand the biochemical properties of human VAPB (hVAPB^WT^) and its ALS-associated P56S mutated form (hVAPB^P56S^), we expressed them in the highly transfectable non-human primate COS-7 cell line. We first investigated their subcellular localization by immunocytochemistry using an antibody specifically raised against the human form of VAPB [17] and antibodies or fluorescent constructs that specifically label compartments of the secretory pathway. We found that hVAPB^WT^ colocalized with the ER marker KDEL (Figure 1A), the COPI vesicle marker β-COP-cyan fluorescent protein (CFP)[23](Figure 1B) and the ER-Golgi intermediate compartment (ERGIC) marker ERGIC-53 (Figure 1C). By contrast, we observed a sparse colocalization of hVAPB^WT^ with Sec23, a marker of COPII vesicles [24](Figure 1D). As previously described [10], [15], [17], [21], the presence of the P56S mutation led to the formation of hVAPB cytoplasmic aggregates. We found that these hVAPB^P56S^ aggregates colocalized with KDEL, β-COP, ERGIC-53 but poorly colocalized with Sec23 in COS-7 cells (Figure 1A–D). We did not observe any co-immunolocalization of hVAPB^WT^ or hVAPB^P56S^ with the Golgi marker GM-130 (data not shown).

**Figure 2:**
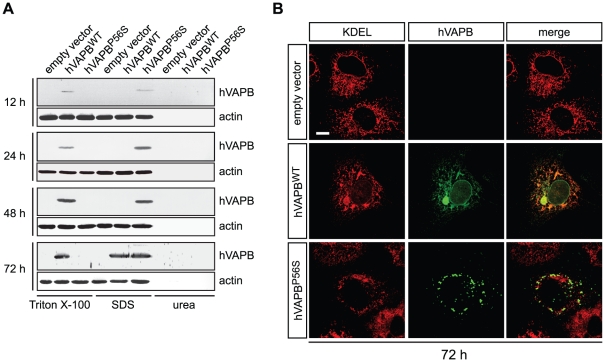
Accumulation of hVAPB^WT^ leads to the formation of cytoplasmic inclusions. (A) Sequential detergent extraction of cellular proteins at different times following transfection of COS-7 cells with indicated an empty vector, hVAPB^WT^ and hVAPB^P56S^ expression vectors. (B) Accumulation of wildtype hVAPB leads to the formation of insoluble inclusions that disrupt ER structure as documented by the co-immunostaining of hVAPB with KDEL 72 h after transfection. Scale bar, 20 µm.

To examine further hVAPB^P56S^ accumulation in COS-7 cells, we conducted differential detergent extraction to analyze its solubility profile. Cells expressing hVAPB^WT^ and hVAPB^P56S^ were first solubilized in a non-ionic detergent (1% Triton X-100) and subjected to high-speed centrifugation. Detergent-resistant fractions were then sequentially extracted in an ionic detergent (1% sodium dodecyl sulfate, SDS) and under chaotropic conditions (8 M urea). Extracted proteins were then separated by SDS-PAGE and analyzed by immunoblotting with hVAPB specific antibodies. We detected the wildtype protein in the Triton X-100 fraction at 12, 24, 48 and 72 h following transfection. At 72 h, we observed that a significant proportion of the wildtype protein was retrieved in the SDS fraction (Figure 2A). When we performed sequential protein extraction on hVAPB^P56S^-expressing COS-7 cells, we found that at all time points, the mutated protein was only found in the SDS-soluble fractions. It is of note that, for both forms of hVAPB, we did not observe the presence of high molecular weight species in the different fractions nor any cleaved fragment (data not shown)[25]. In addition, no hVAPB immunoreactivity was ever found in the urea-soluble fractions over time (Figure 2A). To complement this solubility analysis by immunoblotting, an immunocytochemical study of hVAPB^WT^ at 72 h shows the presence of large cytoplasmic inclusions and a marked disruption of the ER structure, as illustrated by KDEL immunostaining (Figure 2B). At this time of analysis, the immunoreactive profile of hVAPB^P56S^ aggregates was not notably different from earlier time points and no marked disruption of the ER structure was observed (Figure 2B). In conclusion, we confirmed that, in the COS-7 cell line, wildtype hVAPB mainly localizes at the ER and in compartments of the secretory pathway and that the P56S mutation leads to its insolubility and to the formation of cytoplasmic aggregates. Interestingly, in our *in vitro* system, we found that persistent expression of wildtype VAPB also leads to its insolubility and affects ER integrity.

### The ALS-linked P56S mutation decreases the stability of hVAPB

**Figure 3:**
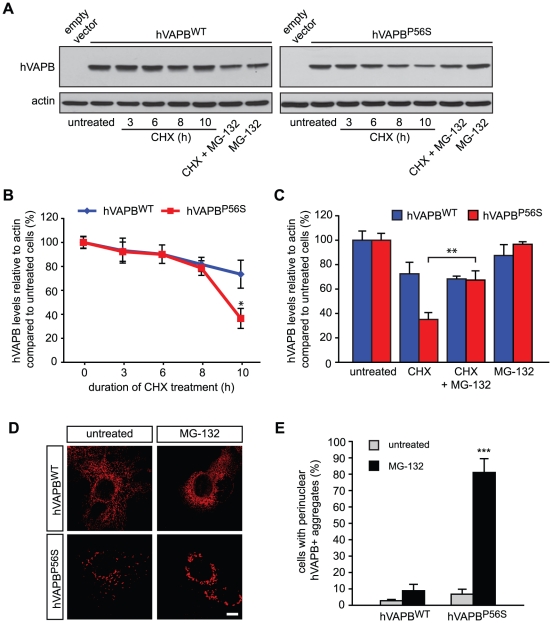
Mutated hVAPB is degraded faster than wildtype hVAPB through a proteasome-dependent mechanism. (A) Western blot analysis of COS-7 cells transfected with the indicated expression vectors and treated or not with the protein biosynthesis inhibitor cycloheximide (CHX, 100 µg/ml) for 3, 6, 8 and 10 h and/or with the proteasome blocking agent MG-132 (10 µM) for 10 h. Actin served as a loading control. (B) hVAPB immunoreactive bands were quantified by densitometry and values were normalized to actin and expressed relative to values obtained in untreated cells. (C) Densitometric quantification of hVAPB levels in transfected cells following 10 h of treatment with CHX and MG-132. (D-E) Immunolabeling of hVAPB in transfected COS-7 cells treated for 12 h with MG-132 (5 µM)(D). The number of cells showing a perinuclear accumulation of hVAPB was determined 36 h after transfection with the indicated vectors (E). Scale bar, 20 µm. Results shown in (B), (C) and (E) are the mean values ± S.D of three independent experiments.

We next studied the stability of both wildtype and mutated VAPB by analyzing their rate of decay following inhibition of neosynthesis by the translational inhibitor cycloheximide (CHX). Transfected cells were treated with CHX for different times before being harvested at 36 h post-transfection and levels of both hVAPB^WT^ and hVAPB^P56S^ were analyzed by Western blotting. We found that after 10 h of CHX treatment, levels of mutated hVAPB were significantly lower than those of the wildtype protein (Figure 3A–C). We were not able to study the effect of a longer treatment due to a significant toxicity of CHX after 10 h (data not shown).

We next asked whether the decreased stability of hVAPB^P56S^ was due to its degradation by the proteasome. Toward this goal, we used the selective proteasome inhibitor MG-132 in combination with CHX in cells transfected with wildtype or mutated hVAPB. Cells were harvested for immunoblot analysis after 10 h of treatment. We found that the addition of MG-132 concomitant with the inhibition of neosynthesis by CHX partially prevented the degradation of hVAPB^P56S^ but had no effect on hVAPB^WT^ levels (Figure 3A, 3C). We confirmed that at this dose, MG-132 efficiently blocks proteasome activity with a limited effect on cell viability (see below). Remarkably, when we analyzed the localization of hVAPB^WT^ and hVAPB^P56S^ by immunocytochemistry, we observed that the inhibition of proteasome activity induced a typical perinuclear accumulation of hVAPB^P56S^ aggregates while the localization of the wildtype protein was not significantly affected (Figure 3D–E). This suggests that proteasome activity partially influences the levels and cytoplasmic scattering of hVAPB^P56S^ aggregates in mammalian cells.

### hVAPB^WT^ and hVAPB^P56S^ increase levels of ubiquitinated conjugates

**Figure 4:**
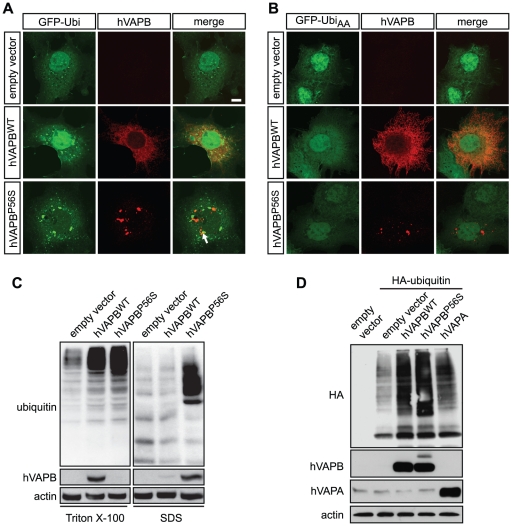
hVAPB^P56S^ partially colocalizes with ubiquitinated conjugates but increases the general ubiquitin levels in the cells. (A) When co-transfected with hVAPB^WT^ or hVAPB^P56S^, GFP-Ubi forms cytoplasmic aggregates that occasionally (white arrow) colocalize with hVAPB^WT^ or hVAPB^P56S^. (B) The GFP-tagged mutated ubiquitin Ubi_AA_-GFP does not form detectable aggregates. Scale bar, 20 µm. (C) Ubiquitin immunoblot profile of COS-7 cells transfected with empty, hVAPB^WT^ and hVAPB^P56S^ vectors following differential detergent extraction. (D) Western blot analysis of total HA-tagged ubiquitin levels in cells expressing either form of hVAPB or hVAPA. In (C) and (D), protein extracts were prepared 36 h after transfection and actin was used as a loading control.

We next sought to examine the ubiquitination profile of mutated hVAPB compared to the wildtype protein. To visualize ubiquitination, we co-expressed either the wildtype or the mutated form of hVAPB with a green fluorescent protein (GFP)-tagged ubiquitin (GFP-Ubi). The GFP-Ubi chimeric protein has been demonstrated to be covalently incorporated into ubiquitin target proteins and to efficiently trace them [26]. When expressed alone, GFP-Ubi reflected the basal ubiquitination process in COS-7 cells with a diffuse cellular fluorescence (Figure 4A). By contrast, when GFP-Ubi was co-expressed with either hVAPB^WT^ or hVAPB^P56S^, we observed an increased accumulation of ubiquitin-positive aggregates that seldom colocalized with hVAPB^WT^ and hVAPB^P56S^ (Figure 4A). As a control, we used a mutated version of the GFP moiety (GFP-Ubi_AA_) that cannot get incorporated into ubiquitinated proteins [26]. Consistently, GFP-Ubi_AA_ failed to form GFP-positive cytoplasmic aggregates both in hVAPB^WT^ and hVAPB^P56S^ expressing cells (Figure 4B). Taken together, these results suggest that wildtype and mutated forms of hVAPB are not major ubiquitination substrates and that an alternative degradation pathway may exist to maintain hVAPB protein homeostasis.

We next examined the detergent solubility features of the ubiquitin conjugates in cells expressing hVAPB^WT^ and hVAPB^P56S^. Differential detergent extraction and Western blot analyzes of ubiquitin first confirmed that both forms of hVAPB caused an increase in the total levels of ubiquitin-conjugated proteins (Figure 4C). However, only the overexpression of mutated hVAPB led to the formation of Triton X-100 insoluble ubiquitin-conjugates. Finally, we evaluated whether the expression of hVAPA, an ER-resident VAP family member that has not been associated with motoneuron disease, leads to a global augmentation of ubiquitin conjugates. Consistent with our previous data, we found that the overexpression hVAPB^WT^ and hVAPB^P56S^ increased the levels of ubiquitinated proteins. By comparison, hVAPA overexpression affected to a lesser extent levels of high molecular weight ubiquitin conjugates (Figure 4D), suggesting that this effect on the overall ubiquitination is preferential to hVAPB.

### Accumulation of hVAPB^WT^ and hVAPB^P56S^ impairs ubiquitin- and ubiquitin-like dependent proteasomal degradation

**Figure 5:**
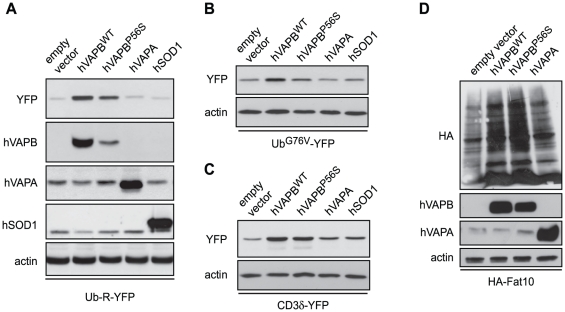
Overexpression of wildtype and mutated hVAPB impairs proteasome activity. Levels of proteasome YFP reporters in cells co-transfected (or not) for 36 h with hVAPB^WT^, hVAPB^P56S^, hVAPA, hSOD1 and Ub-R-YFP (A), Ub-G76V-YFP (B) or CD3δ-YFP (C) were examined by Western blotting using anti-GFP antibodies. (D) Cells were co-transfected with vectors encoding hVAPB^WT^, hVAPB^P56S^ or hVAPA and HA-tagged Fat10, an ubiquitin-independent signal for proteasomal degradation. Protein extracts were prepared 36 h post-transfection, resolved by SDS-PAGE, Western blotted and probed with HA, hVAPB, hVAPA and actin antibodies.

We next investigated whether the elevated levels of ubiquitin-protein conjugates observed following hVAPB^WT^ and hVAPB^P56S^ expression involve an impairment of proteasomal degradation. Toward this goal, we took advantage of some well-established yellow fluorescent protein (YFP)-tagged specific substrates that, when expressed in living cells, are degraded by the proteasome through different pathways [27]. Namely, these substrates are Ub-R-YFP, a short-living cytosolic substrate degraded through the N-end rule pathway [27], [28]. Ub^G76V^-YFP, a cytosolic substrate degraded via the ubiquitin fusion degradation (UFD) system [27], [29] and CD3δ-YFP, a T-cell receptor subunit whose degradation occurs through the ER-associated protein degradation (ERAD) system, which translocates misfolded protein from the ER to the cytosol for degradation [30], [31]. We determined the proteolytic activity of the proteasome through Western blot analysis of YFP levels in cells transiently co-transfected with plasmids driving expression of the different proteasome substrates and either hVAPB^WT^ or hVAPB^P56S^. The overexpression of wildtype and mutated hVAPB increased the steady-state levels of the three proteasome reporters Ub-R-YFP, Ub^G76V^-YFP and CD3δ-YFP (Figure 5A–C). To ensure that the increased accumulation of the proteasome substrates was not due to an unspecific effect of protein overload, we co-expressed hVAPA or the human superoxide dismutase-1 (hSOD1) with the different proteasome reporters. In these cases, we observed that neither hVAPA nor hSOD1 overexpression led to an increase in proteasome substrates as marked as both wildtype or mutated hVAPB did (Figure 5A–C). These data suggest that overexpression of both wildtype and mutated forms of hVAPB impair UPS independently of the pathway leading to proteasomal degradation (N-end rule, UFD or ERAD).

We next asked whether the negative effect of hVAPB on proteasome activity was restricted to proteins that depend on ubiquitin signal for their degradation. F-adjacent transcript-10 (Fat10) is an ubiquitin-like protein that serves as a signal for degradation by the proteasome [32]. A HA-tagged Fat10 was then co-expressed with hVAPB^WT^, hVAPB^P56S^ or hVAPA. We found that co-expression of HA-Fat10 with either hVAPB^WT^ or hVAPB^P56S^ led to an increased accumulation of total HA-Fat10-conjugated proteins compared to a control empty vector or a vector expressing hVAPA (Figure 5D). These results indicate that overexpression of wildtype or mutated hVAPB can impair proteasome activity independently of the targeting signal.

### ER stress contributes to hVAPB-induced impairment of the proteasome activity

**Figure 6:**
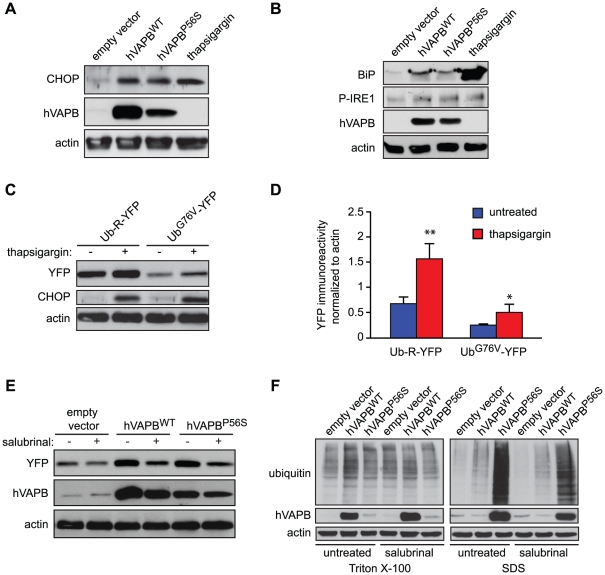
hVAPB-mediated ER stress contributes to the accumulation of proteasome substrates. (A) The immunoreactivity of CHOP in cells expressing hVAPB^WT^ and hVAPB^P56S^ was monitored by Western blotting (36 h after transfection). Thapsigargin treatment (for 16 h) was used as a positive control for ER stress-dependent CHOP upregulation. (B) Levels of BiP and phosphorylation status of IRE1 were examined by Western blotting 36 h following the transfection of cells with empty, hVAPB^WT^ and hVAPB^P56S^ plasmids. (C) Protein extracts of cells transfected with the proteasome reporters Ub-R-YFP and Ub^G76V^-YFP and treated (or not) for 16 h with the ER stress inducer thapsigargin (10 µM) were subjected to Western blotting using anti-GFP (referred to as YFP), and anti-CHOP antibodies. (D) Quantification of the YFP immunoreactive bands (C) normalized to actin signals (arbitrary densitometry units). (E) Salubinal treatment (20 µm) diminished the accumulation of the proteasome reporter (YFP) as indicated by Western blotting of COS-7 cells co-transfectd with hVAPB^WT^ or hVAPB^P56S^. (F) Differential detergent extraction and Western blot analysis of ubiquitin in cells expressing hVAPB^WT^ and hVAPB^P56S^ and treated or not with salubrinal (20 µM).

We have recently demonstrated that adeno-associated virus (AAV)-mediated expression of wildtype and mutated hVAPB in motoneurons leads to an ER stress response that contributes to neurodegeneration [17]. It has been shown that ER stress may impede UPS activity [30]. This prompted us to examine whether the impairment of proteasome activity following hVAPB^WT^ or hVAPB^P56S^ overexpression is caused by ER stress. We first evaluated whether overexpression of hVAPB^WT^ and hVAPB^P56S^ elicits an ER stress response in COS-7 cells by analyzing the induction of the ER stress marker C/EBP-homologous protein (CHOP)[33]. As depicted in Figure 6A, we observed a marked increase of CHOP protein in cells expressing wildtype and mutated hVAPB. Furthermore, we show that the overexpression of both wildtype and mutated hVAPB leads to the induction of the immunoglobulin binding protein, BiP, as well as an increased phosphorylation of the inositol-requiring enzyme 1 (IRE1); two ER stress markers [34], [35](Figure 6B). We next evaluated whether ER stress compromises UPS activity in COS-7 cells by analyzing the proteasome reporter (Ub-R-YFP and Ub^G76V^-YFP) levels. We found that ER stress impairs proteasome activity as shown by the elevated levels of proteasome substrates following treatment with the ER stress inducer thapsigargin in our experimental conditions (Figure 6C–D).

We then sought to examine the effect of the ER stress inhibitor salubrinal on proteasome activity following hVAPB^WT^ and hVAPB^P56S^ overexpression [36]. We first ensured that the dose of salubrinal we used prevented the increase phosphorylation of eukaryotic translation initiation factor 2 subunit alpha under stress condition in COS-7 cells (data not shown). We then found that salubrinal significantly decreased by 42% and 31% the accumulation of proteasome substrates following overexpression of hVAPB^WT^ and hVAPB^P56S^ respectively (Figure 6E). To further confirm that hVAPB-mediated ER stress impairs proteasome activity, we analyzed the overall profile of ubiquitination by differential detergent extraction and Western blot with anti-ubiquitin antibodies in COS-7 expressing either form of hVAPB in the presence of salubrinal. Consistently, we observed that salubrinal decreased the elevated levels of ubiquitin-conjugates in both Triton X-100 and SDS fractions that accompanied the overexpression of hVAPB^WT^ and hVAPB^P56S^ (Figure 6F). These results suggest that ER stress contributes to the impairment of UPS activity induced by overexpression of both wildtype and mutated form of hVAPB, though other mechanisms might also exist.

### Wildtype and mutated hVAPB sequester the proteasome

**Figure 7:**
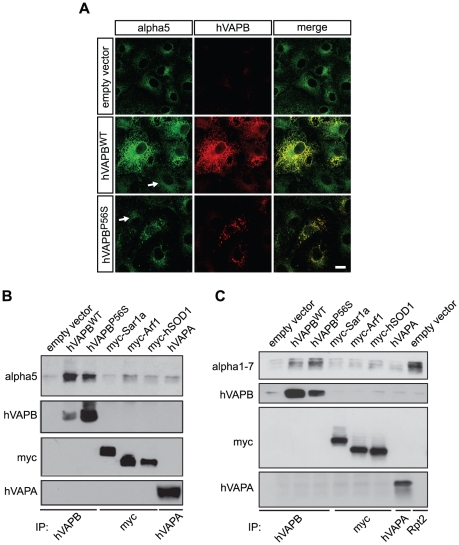
Wildtype and mutated hVAPB associate with the proteasome. (A) Both hVAPB^WT^ and hVAPB^P56S^ colocalize with the alpha 5 subunit of the proteasome, as indicated by the double immunostaining of cells transfected with empty, hVAPB^WT^ and hVAPB^P56S^ vectors. Images were acquired with the same exposure time and camera settings. White arrows indicates non-transfected cells. Scale bar, 20 µm. (B–C) hVAPB^WT^ and hVAPB^P56S^ were immunoprecipitated from COS-7 cells transfected with hVAPB^WT^, hVAPB^P56S^, myc-tagged Sar1, myc-tagged Arf1, myc-tagged hSOD1 and hVAPA. Endogenous alpha 5 (B) or alpha 1–7 (C) subunits of the proteasome that co-immunoprecipitated with hVAPBs was detected by Western blotting using specific antibodies. hVAPB, myc (Sar1, Arf1 and hSOD1) and hVAPA input levels are shown. Immunoprecipitation of alpha 1–7 by Rpt2 served as a positive control.

It has been observed in SOD1 mutant mouse spinal cords that the 20S proteasome particle was trapped in neuronal inclusions [37]. We therefore asked whether this histological characteristic was also shared by another ALS-causing gene and whether the proteasome could be complexed by hVAPB. We performed an immunostaining analysis of the alpha 5 subunit of the proteasome in COS-7 cells expressing wildtype or mutated hVAPB. Interestingly, we found that the proteasome was markedly retained at the ER in cells overexpressing wildtype hVAPB, and it was also found sequestered in hVAPB^P56S^ cytoplasmic aggregates (Figure 7A). This observation suggests that hVAPB might bind to the proteasome, either directly or indirectly.

We performed an immunoprecipitation assay of the endogenous alpha 5 subunit from COS-7 cells expressing hVAPB^WT^, hVAPB^P56S^ or other proteins used as controls: the COPII coat protein Sar1, the COPI coat protein ADP-ribosylation factor 1 (Arf1), as well as hSOD1 and hVAPA. We found that both wildtype and mutated hVAPB immunoprecipitated endogenous alpha 5, whereas alpha 5 was not found in Sar1, Arf1, hSOD1 or hVAPA immunoprecipitates (Figure 7B). We did not perform reverse immunoprecipitation experiments, consisting in the immunoprecipitation of alpha 5 and the immunodetection of hVAPB, since hVAPB^P56S^ aggregates were precipitated by low centrifuge force (as low as 500×*g*) independently of sepharose bead-conjugated immunocomplexes (data not shown). Therefore, we conducted an immunoprecipitation of hVAPB followed by an immunoblotting analysis using an antibody that recognizes several alpha subunits (alpha 1–7) of the 20S proteasome particle. Consistently, we observed that both hVAPB^WT^ and hVAPB^P56S^ were able to co-immunoprecipitate the proteasome (Figure 7C). By contrast, neither Sar1, Arf1, hSOD1 nor hVAPA was found to efficiently immunoprecipitate the proteasome. As a positive control, we observed that Rpt2, a subunit of the 19S regulatory complex of the proteasome, efficiently co-immunoprecipitated the alpha subunits of the 20S proteasome core particle (Figure 7C).

However, when hVAPB^WT^ and hVAPB^P56S^ expressing cells were subjected to hVAPB immunoprecipitation, we failed to observe immunoreactivity for the 19S proteasome subunit Rpt2 or Rpn10 in the precipitates (data not shown). Altogether, these results show that both wildtype and mutated hVAPB can be found in association with the 20S proteasome particle.

## Discussion

The pathology of ALS includes a defective protein homeostasis and the formation of protein aggregates. This led us to study whether the overexpression of both wildtype and mutated hVAPB, previously demonstrated to selectively triggers death of motoneurons [17], interferes with protein turnover. We found that the overexpression of both wildtype and mutated hVAPB impairs proteasome function and increases the burden of ubiquitin- and ubiquitin-like conjugates. Our data show that an ER stress response elicited by the forced expression of both hVAPB^WT^ and hVAPB^P56S^ contributes to the disruption of proteasome activity. In addition, we found that both wildtype and mutated hVAPB interact with the 20S proteasome, providing a potential mechanism of UPS impairment.

Protein aggregates have already been described in cellular and animal models of ALS-linked to hVAPB. An accumulation of ubiquitin-positive aggregates that colocalize to a minor degree with TAR DNA-binding protein (TDP-43)-positive hVAPB^P56S^ aggregates was documented in spinal motoneurons of hVAPB mutant transgenic mice [22]. In HeLa cells, hVAPB^P56S^ aggregates were found to be poorly ubiquitinated [21]. A similar discordant distribution of ubiquitin and hVAPB aggregates was documented for the two P56S and T46I mutated forms of hVAPB in NSC34 cells [18]. This is consistent with our analysis, which shows that the mutated form of hVAPB led to the formation of ubiquitin aggregates and which seldom colocalized with mutated hVAPB (Figure 4A). Several hypotheses could explain this partial colocalization between hVAPB and ubiquitin aggregates. A proportion of these ubiquitinated proteins could include hVAPB binding partners [38], or represent a transient aggregation of ubiquitinated hVAPB, targeted to proteasomal clearing. The abortive exit of hVAPB mutant from the ER could indeed leads to its degradation [39]. Accordingly, we found that mutated hVAPB is degraded faster than the wildtype form and that proteasome inhibition partially prevents hVAPB^P56S^ from degradation following cycloheximide treatment (Figure 3C). Of note, many ALS-causing mutations have also been shown to destabilize SOD1 protein [40]. Consistent with previous data [15], when we carried out an immunoprecipitation of both forms of hVAPB in COS-7 cells co-expressing a haemagglutinin (HA)-tagged ubiquitin [41], several ubiquitin-positive bands at higher molecular weight were detected (data not shown). It is noteworthy that an increased global ubiquitination profile was also observed in cells expressing the wildtype form of hVAPB, though to a lesser extent that the mutated hVAPB [15]. However, we could not rigorously evaluate whether the high-molecular-weight species labeled with ubiquitin represent poly-ubiquitinated forms of hVAPB or mono- and poly-ubiquitinated hVAPB binding proteins.

Our data suggest that the degradation of mutated as well as wildtype hVAPB also occur through alternative mechanisms independent of the ubiquitin proteasome system. Indeed, it has been shown that both wildtype and ALS-associated mutated forms of SOD1 can be degraded by both the proteasomal and autophagy pathways [42]. Nevertheless, Teuling, E and colleagues did not observe any colocalization of autophagy-related (Atg)-8 (LC3) and Atg12 proteins with hVAPB^P56S^ aggregates [21]. Consistently, we did not observe any significant co-localization of hVAPB^P56S^ aggregates with the autophagosomal marker LC3, nor the cleavage and activation of LC3 by Western blot in our experimental conditions. In addition, stimulation of autophagy in cells expressing hVAPB^P56S^ by rapamycin did not significantly decrease levels of SDS-soluble aggregates (data not shown). Other options for protein degradation have been demonstrated to modulate levels of pathogenic proteins. For instance, the insulin-degrading enzyme and neprilysin are two metalloproteases that contribute to amyloid β-peptide degradation [43], which therefore represents attractive therapeutic targets for Alzheimer’s disease [44]. Another intriguing possibility relates the role of the 20S proteasome in the selective recognition and degradation of oxidized proteins in an ubiquitin-independent manner [45]. Oxidative stress plays an important role in ALS pathogenesis and reactive oxygen and nitrogen species might contribute to hVAPB modification. The use of proteome technologies will be pivotal to explore the precise post-translational modifications of hVAPB.

Two dynamic-quality control compartments have been described which serve as deposit for misfolded proteins. A dynamic deposit site, the juxta nuclear quality control (JUNQ), contains ubiquitinated proteins and an insoluble protein deposite (IPOD) site, which incorporates immobile and non-ubiquitinated proteins [46]. Quantitative fluorescence recovery after photobleaching experiments demonstrated that the mutant hVAPB^P56S^ forms immobile cytosolic aggregates [21]. We could speculate that the preparation of hVAPB^P56S^ for clearing occurs in IPOD centers. However, IPOD compartments colocalize with autophagic marker (that we and others did not find associated with mutated hVAPB aggregates) and do not physically interact with the proteasome (that we demonstrated to be consistently trapped into hVAPB mutant aggregates). Thus, it is possible that hVAPB mutant aggregates are directed to other compartments for their degradation. Collectively, these results underline the importance of studying the proteolytic machinery that control hVAPB levels.

Next, we focused our study on how hVAPB buildup might cause UPS impairment. Recently, we found that the viral-promoted expression of both wildtype and mutated hVAPB selectively triggered death of motoneurons. It was intriguing that both forms of hVAPB elicited an ER stress response, as examined by the quantification of fluorescent intensity of phosphorylated IRE1 immunolabelling, in embryonic motoneurons. The functional involvement of ER stress in motoneuron death was illustrated by the neuroprotective effect of salubrinal against AAV-mediated overexpression of hVAPB^WT^ and hVAPB^P56S^ [17]. ER stress, which was demonstrated to compromise UPS, was of a particular interest [30]. Unfortunately, the limited quantities of materials obtained from primary culture of motoneurons preclude many biochemical studies. Here, we confirmed that in non-human primate cells the expression of hVAPB^WT^ and hVAPB^P56S^ led to ER stress (Figure 6A–B), giving us the opportunity to investigate the contribution of ER stress to the impairment of UPS function. It has previously been demonstrated that ER stress, elicited by thapsigargin or tunicamycin, impairs degradation of UPS reporter substrates, although ER stressors induce a modest rather than a complete impairment of UPS functions [30]. Correspondingly, we found that the overexpression of hVAPB^WT^ and hVAPB^P56S^, which leads to the robust induction of the ER stress marker CHOP, BiP and phosphorylation of IRE1, induce an accumulation of UPS reporter substrates. However, the inhibition of ER stress by salubrinal did partially abrogate UPS impairment (Figure 6E–F). Since our work reveals that an ALS-causing gene impairs proteasome function partially through the ER stress pathway, it is conceivable that the partial but long-lasting impairment of proteasome function subsequent to a chronic ER stress contributes to the progressive and pernicious accumulation of proteins. The mechanisms by which ER stress leads to proteasome dysfunction remains to be examined in future studies. It has been demonstrated that an ER stress response take place early in the history of the disease, well before the first clinical symptoms and the administration of salubrinal in SOD1 mutant mice conferred therapeutic benefit [47]. From the same study, it was found that an augmentation of ubiquitin signals in ALS-vulnerable as well as resistant motoneurons shortly precedes a marked ER stress response that selectively takes place in the ALS-vulnerable population. It is conceivable that the impairment of proteasome activity elicited by the accumulation of hVAPB^WT^ and hVAPB^P56S^ precedes the ER stress response. However, our data also suggest that the ER stress induced by the overexpression of wildtype and mutated hVAPB contribute to the impairment of UPS activity (Figure 6), which can in turn elicits an ER stress response. An amplification process that would selectively manifest in vulnerable motoneurons prone to ER stress [47], could contribute to ALS pathogenesis. A better understanding of ER stress signaling will therefore be instrumental for the development of effective therapies for motoneuron disease as well as other conformational disorders including Alzheimer’s and Parkinson’s disease, in which ER stress also play an important role [48].

Our results also provide evidence that mutated hVAPB interacts and partitions the 20S proteasome into cytoplasmic aggregates (Figure 7A–C). This may indicate that a limited availability of the proteasome caused by its sequestration into aggregates could lead to the accumulation of ubiquitin and Fat10 conjugates (Figure 4C–D and 5D). Neurodegenerative disorders have been reported to be associated with accumulation of proteasome into neuronal inclusions. For example, the 20S proteasome localize at the site of mutant Ataxin-1 aggregation in neurons of spinocerebellar ataxia type 1 patients [49]. In the brain of Lafora disease patients, a recruitment of the 20S proteasome has been observed in polyglucosan aggregates [50]. Of particular relevance to our study, a focal and intense accumulation of 20S proteasome was observed in spinal motoneurons of sporadic ALS patients [51]. Ubiquitin-positive aggregates can be hypothesized as a challenging substrate for the proteasome, leading therefore to an impairment of the proteolytic machinery. However, in our study, ubiquitinated aggregates poorly colocalize with mutated hVAPB, while the 20S proteasome is mainly associated with hVAPB^P56S^ aggregates. This suggests that the trapping of the proteasome by mutated hVAPB would occur independently of its ubiquitination. Consistent with this, the wildtype form of hVAPB interacts with the proteasome and leads to its retention at the ER (Figure 7A–C), and this could compromise its activity resulting in the accumulation of ubiquitin- or Fat10–conjugated proteins. We observed that the overexpression of wildtype hVAPB led to the accumulation of the ERAD substrate CD3δ (Figure 5C). CD3δ-YFP, as well as the two others cytosolic substrates, allow us to show an impairment of the proteosomal activity. However, we cannot rule out the possibility that the ERAD machinery, which mediates the translocation of misfolded proteins from the ER to the cytoplasm for their subsequent degradation by the UPS, is also compromised [52]. Inhibition of ERAD by SOD1 mutant, through its interaction with Derlin-1, a functional component of the ERAD, has been proposed to trigger ER stress [53]. An impairment of ERAD function by hVAPB would contribute to the accumulation of misfolded proteins in the ER, eliciting therefore an ER stress response. As discussed above, such a feedback amplification mechanism would progressively lead a more global disturbance of protein homeostasis.

Biogenesis of the proteasome, which is a highly ordered multistep process implicating several assembly and maturation factors, mainly takes place at the ER [54], [55]. An intriguing working hypothesis is that hVAPB interacts with the assembly machinery at the ER to contribute to proteasome formation. The abnormal presence of wildtype hVAPB as well as the mutated protein, as long as it retains its ability to insert into the ER membrane and form cytoplasmic inclusion continuous with the ER [19], might interfere in the biogenesis program. This could explain the neurotoxicity observed in motoneurons following the overexpression of both wildtype and mutated hVAPB [17]. It would be interesting to examine whether hVAPB and other ALS-causing gene products modify the assembly and activation program of the proteasome in motoneurons.

## Materials and Methods

### Cell culture

COS-7 cells (American Type Culture Collection, ATCC, Manassas, VA, USA) were cultured in 10 cm diameter dishes for immunoprecipitation experiments, in 6-well plates for Western blot analysis or on 12 mm diameter glass coverslips (placed in 24-well plates) for immunostaining. Cells were maintained in Dulbecco Modified Eagle Medium (DMEM, Invitrogen, Carlsbad, CA, USA) supplemented with 10% fetal bovine serum and penicillin (50 U/ml) and streptomycin (50 µg/ml)(Invitrogen). Cells were plated in different culture dishes at the density of 20,000 cell per cm^2^, transfected the day after with 10 µg of indicated expression vector for 10 cm diameter dishes, 3 µg for 6-well plates and 0.8 µg for 24-well plates using Fugene 6 transfection reagent following the manufacturer’s instruction (Roche diagnostics, Indianapolis, IN, USA). An equimolar ratio was used for co-transfection experiments. We transfected corresponding empty expression vectors for control samples. We reproducibly found by PCR analysis our cell line to be free of mycoplasma contamination.

### Reagents

Cycloheximide and MG-132 were purchased from Sigma-Aldrich (St Louis, MO, USA), salubrinal was purchased from Enzo life sciences (Lausen, Switzerland, USA) and thapsigargin from Millipore (Calbiochem, Millipore, Bedford, MA, USA).

### Expression vectors

hVAPB^WT^, hVAPB^P56S^ and hVAPA coding sequences were placed under the control of the human ubiquitin c promoter as we previously described (pUbc-hVAPB^WT^, pUbc-hVAPB^P56S^ and pUbc-hVAPA)[17]. The Arf1 coding sequence was amplified from mouse spinal cord using the following sense primer 5’-ACCATGGGGAATATCTTTGCAAACCTC-3’ and antisense primer: 5’-CTTCTGGTTCCGGAGCTGATTAGAC-3’ as previously described [56]. The amplified sequence was cloned into the pCR2.1 vector (Invitrogen). Arf1 cDNA was then incorporated in frame with a myc tag sequence into the pCS2+-myc expression vector. The probity of the sequence was confirmed by sequencing. The myc-Sar1 (pCS2+-myc-Sar1) expression vector has been described in [56].

β-COP-CFP construct was kindly provided by Irina Majoul [23], Sec23-YFP by David Stephens [24], GFP-Ubi and GFP-UbiAA by Michel Bouvier [26], HA-ubiquitin by Simone Diestel [41], HA-Fat10 by Marcus Groettrup [32]. Ub-R-YFP, Ub^G76V^-YFP and CD3δ-YFP constructs were obtained from Addgene (Cambridge, MA, USA). Myc-hSOD1 expression vector was obtained by cloning hSOD1 coding sequence [57]. All plasmids (at the exception of hVAPB^WT^, hVAPB^P56S^ and hVAPA as mentioned above), constitutively express the indicated cDNA from the cytomegalovirus (CMV) promoter.

### Immunocytochemistry

Immunofluorescence staining was performed as we previously described [17], [58]. Briefly, cells were cultured onto glass coverslips and fixed in 3.7% formaldehyde for 20 min at room temperature (RT). After three washes in phosphate-buffered saline (PBS), cells were incubated for 1 h in PBS containing 0.1% Triton X-100, 4% bovine serum albumin (BSA) and 5% heat-inactivated donkey serum (blocking solution). Cells were incubated overnight at 4°C in the blocking solution containing the following primary antibodies: anti-KDEL (10C3, Enzo life sciences, 1∶400), anti-ERGIC-53/p58 (E1031, Sigma-Aldrich, 1∶200), anti-GM130 (612008, BD Biosciences, Franklin Lakes, NJ, USA, 1∶300) anti-alpha5 proteasome subunit (MCP196, Enzo life sciences, 1∶250). Cells were washed 4 times for 5 min each with PBS, incubated with the appropriate fluorescent-conjugated secondary antibody (Invitrogen), washed, and mounted in moviol containing 1,4-diazobicyclo-[2.2.2]-octane (DABCO, Sigma-Aldrich). Images were collected using either an Olympus BX50WI laser-scanning confocal or a Zeiss ApoTome microscope.

### Differential detergent extraction

The cells were washed with ice-cold PBS, scraped and centrifuged at 1,500×*g* for 5 min at +4°C. Cell pellet was first resuspended in 50 mM Tris-HCl pH 7.5, 150 mM NaCl, 2 mM EDTA, 2 mM EGTA and 1% Triton X-100 and incubated on ice for 20 min. Cells were centrifuged at 120,000×*g* for 30 min at 4°C. The collected supernatant constituted the Triton X-100-soluble fraction. The pellet was then resuspended in 50 mM Tris-HCl pH 7.5, 150 mM NaCl, 2 mM EDTA, 2 mM EGTA and 1% SDS and centrifuged at 120,000×*g* for 30 min at 4°C, in order to collect the supernatant, referred to as the SDS fraction. Finally, the pellet was resuspended in urea lysis buffer (50 mM Tris-HCl pH 7.5, 150 mM NaCl, 2 mM EDTA, 2 mM EGTA and 8 M urea) and constituted the urea-soluble fraction.

### Western blotting

Western blotting was carried out on cell lysates using the protocol previously described [17], [58]. Otherwise indicated, total proteins were extracted using SDS lysis buffer (50 mM Tris-HCl pH 7.5, 150 mM NaCl, 2 mM EDTA, 2 mM EGTA and 1% SDS) supplemented with a protease inhibitor cocktail (Roche diagnostics). Protein concentration was determined using the BCA kit following manufacturer’s instructions (Pierce, Rockford, IL, USA). After a 5 min denaturation step at 95°C in Laemmli buffer (62.5 mM Tris-HCl pH6.8; 2% SDS; 10% glycerol; 2.5% β-mercaptoethanol; 0.0075% bromophenol blue), protein samples were separated by sodium dodecyl sulfate polyacrylamide gel electrophoresis (SDS-PAGE) and blotted to nitrocellulose membranes (Schleicher and Schuell, Whatman International Ltd, Springfield Mill, UK). The membranes were washed with PBS containing 0.1% Tween-20 (PBST) and blocked in PBST with 5% non-fat dry milk. The following primary antibodies were diluted in PBST with 3% BSA at concentrations and applied overnight at +4°C: anti-hVAPB (DIM705; previously described in [17]; 1∶2,000), anti-actin (AC-40; Sigma-Aldrich; 1∶4,000), anti-ubiquitin (Z0458; DAKO, Glostrup, Denmark; 1∶1,000), anti-HA (16B12; Covance; 1∶1,000), anti-GFP (TP401; Torrey Pines Biolabs, East Orange, NJ, USA; 1∶2,000), anti-VAPA (K15; Santa Cruz Biotechnology, Santa Cruz, CA, USA; 1∶2,000), anti-SOD1 (574597; Calbiochem; 1∶2,000), anti-myc (9E10; SantaCruz Biotechnologies; 1∶2,000), anti-CHOP (F-168; Santa Cruz Biotechnologies; 1∶1,000), anti-BiP (3177, Cell Signaling Technology, 1∶1,000), anti-phospho-IRE1 (ab48187, Abcam, Cambridge, MA, USA, 1∶1,000), anti-alpha5 (MCP196; Enzo life sciences; 1∶1,000) and anti-alpha1-7 (MCP231; Enzo life sciences; 1∶1,000). Proteins were then detected using horseradish peroxidase-conjugated secondary antibodies (DAKO), chemiluminescent horseradish peroxidase substrate (Millipore) and X-MR films (Kodak). When required, immunoblot images were quantified by densitometric analysis of immunoreactive bands using the ImageJ software (National Institutes of Health, USA) and normalized relative to their respective actin signals.

### Immunoprecipitation

Cells were washed twice with cold PBS and lysed on ice for 10 min in IP buffer (20 mM Tris-HCl pH 7.5, 150 mM NaCl, 5 mM EDTA, 5 mM EGTA, 2 mM ATP, 10% glycerol and 1% Triton X-100) with proteases inhibitors. Lysates were pre-cleared by a 5 min centrifugation at 100×*g* and then protein concentration was determined by a modified Bradford reaction (Bio-rad laboratories, Hercules, CA, USA). 1 mg of total proteins (adjusted to a volume of 500 µl with IP buffer) were incubated overnight at +4°C with appropriate primary antibodies: hVAPB (1∶100), hVAPA (1∶500), myc (1∶500), Rpt2 (1∶250) and Protein A or G sepharose beads (20 µl packed beads, GE Healthcare, Buckinghamshire, UK). Immunocomplexes were collected by centrifugation for 1 min at 1,000×*g* at +4°C. After three rounds of washes with 800 µl of IP buffer each, the immunoprecipitated proteins were denatured 5 min at 95°C in Laemmli buffer and resolved by SDS-PAGE as described for Western blotting.

### Statistical analyzes

Data represent the mean values ± standard deviation (S.D) of at least three independent experiments, each done at least in duplicate. Statistical significance was determined by an unpaired two-tailed Student’s *t* test using GraphPad Prism (GraphPad Software, La Jolla, CA, USA). Significance was accepted at the level of *P* < 0.05. *P* values are expressed as *P < 0.05, **P < 0.01 and ***P < 0.001.

## References

[1] Strong MJ, Kesavapany S, Pant HC (2005). The pathobiology of amyotrophic lateral sclerosis: a proteinopathy?. J Neuropathol Exp Neurol.

[2] Wood JD, Beaujeux TP, Shaw PJ (2003). Protein aggregation in motor neurone disorders.. Neuropathol Appl Neurobiol.

[3] Hochstrasser M (2009). Origin and function of ubiquitin-like proteins.. Nature.

[4] Cheroni C, Peviani M, Cascio P, Debiasi S, Monti C (2005). Accumulation of human SOD1 and ubiquitinated deposits in the spinal cord of SOD1G93A mice during motor neuron disease progression correlates with a decrease of proteasome.. Neurobiol Dis.

[5] Kabashi E, Agar JN, Taylor DM, Minotti S, Durham HD (2004). Focal dysfunction of the proteasome: a pathogenic factor in a mouse model of amyotrophic lateral sclerosis.. J Neurochem.

[6] Deng HX, Zhai H, Bigio EH, Yan J, Fecto F (2010). FUS-immunoreactive inclusions are a common feature in sporadic and non-SOD1 familial amyotrophic lateral sclerosis.. Ann Neurol.

[7] Tagawa A, Tan CF, Kikugawa K, Fukase M, Nakano R (2007). Familial amyotrophic lateral sclerosis: a SOD1-unrelated Japanese family of bulbar type with Bunina bodies and ubiquitin-positive skein-like inclusions in lower motor neurons.. Acta Neuropathol.

[8] Piao YS, Wakabayashi K, Kakita A, Yamada M, Hayashi S (2003). Neuropathology with clinical correlations of sporadic amyotrophic lateral sclerosis: 102 autopsy cases examined between 1962 and 2000.. Brain Pathol.

[9] Neumann M, Sampathu DM, Kwong LK, Truax AC, Micsenyi MC (2006). Ubiquitinated TDP-43 in frontotemporal lobar degeneration and amyotrophic lateral sclerosis.. Science.

[10] Nishimura AL, Mitne-Neto M, Silva HC, Richieri-Costa A, Middleton S (2004). A mutation in the vesicle-trafficking protein VAPB causes late-onset spinal muscular atrophy and amyotrophic lateral sclerosis.. Am J Hum Genet.

[11] Soussan L, Burakov D, Daniels MP, Toister-Achituv M, Porat A (1999). ERG30, a VAP-33-related protein, functions in protein transport mediated by COPI vesicles.. J Cell Biol.

[12] Peretti D, Dahan N, Shimoni E, Hirschberg K, Lev S (2008). Coordinated lipid transfer between the endoplasmic reticulum and the Golgi complex requires the VAP proteins and is essential for Golgi-mediated transport.. Mol Biol Cell.

[13] Amarilio R, Ramachandran S, Sabanay H, Lev S (2005). Differential regulation of endoplasmic reticulum structure through VAP-Nir protein interaction.. J Biol Chem.

[14] Gkogkas C, Middleton S, Kremer AM, Wardrope C, Hannah M (2008). VAPB interacts with and modulates the activity of ATF6..

[15] Kanekura K, Nishimoto I, Aiso S, Matsuoka M (2006). Characterization of amyotrophic lateral sclerosis-linked P56S mutation of vesicle-associated membrane protein-associated protein B (VAPB/ALS8).. J Biol Chem.

[16] Suzuki H, Kanekura K, Levine TP, Kohno K, Olkkonen VM (2009). ALS-linked P56S-VAPB, an aggregated loss-of-function mutant of VAPB, predisposes motor neurons to ER stress-related death by inducing aggregation of co-expressed wild-type VAPB.. J Neurochem.

[17] Langou K, Moumen A, Pellegrino C, Aebischer J, Medina I (2010). AAV-mediated expression of wild-type and ALS-linked mutant VAPB selectively triggers death of motoneurons through a Ca2+-dependent ER-associated pathway.. J Neurochem.

[18] Chen HJ, Anagnostou G, Chai A, Withers J, Morris A (2010). Characterization of the properties of a novel mutation in VAPB in familial amyotrophic lateral sclerosis.. J Biol Chem.

[19] Fasana E, Fossati M, Ruggiano A, Brambillasca S, Hoogenraad CC (2010). A VAPB mutant linked to amyotrophic lateral sclerosis generates a novel form of organized smooth endoplasmic reticulum.. Faseb J.

[20] Prosser DC, Tran D, Gougeon PY, Verly C, Ngsee JK (2008). FFAT rescues VAPA-mediated inhibition of ER-to-Golgi transport and VAPB-mediated ER aggregation.. J Cell Sci.

[21] Teuling E, Ahmed S, Haasdijk E, Demmers J, Steinmetz MO (2007). Motor neuron disease-associated mutant vesicle-associated membrane protein-associated protein (VAP) B recruits wild-type VAPs into endoplasmic reticulum-derived tubular aggregates.. J Neurosci.

[22] Tudor EL, Galtrey CM, Perkinton MS, Lau KF, De Vos KJ (2010). Amyotrophic lateral sclerosis mutant vesicle-associated membrane protein-associated protein-B transgenic mice develop TAR-DNA-binding protein-43 pathology.. Neuroscience.

[23] Majoul I, Straub M, Hell SW, Duden R, Soling HD (2001). KDEL-cargo regulates interactions between proteins involved in COPI vesicle traffic: measurements in living cells using FRET.. Dev Cell.

[24] Watson P, Forster R, Palmer KJ, Pepperkok R, Stephens DJ (2005). Coupling of ER exit to microtubules through direct interaction of COPII with dynactin.. Nat Cell Biol.

[25] Gkogkas C, Wardrope C, Hannah M, Skehel P (2011). The ALS8-associated mutant VAPB(P56S) is resistant to proteolysis in neurons.. J Neurochem.

[26] Perroy J, Pontier S, Charest PG, Aubry M, Bouvier M (2004). Real-time monitoring of ubiquitination in living cells by BRET.. Nat Methods.

[27] Dantuma NP, Lindsten K, Glas R, Jellne M, Masucci MG (2000). Short-lived green fluorescent proteins for quantifying ubiquitin/proteasome-dependent proteolysis in living cells.. Nat Biotechnol.

[28] Bachmair A, Finley D, Varshavsky A (1986). In vivo half-life of a protein is a function of its amino-terminal residue.. Science.

[29] Johnson ES, Ma PC, Ota IM, Varshavsky A (1995). A proteolytic pathway that recognizes ubiquitin as a degradation signal.. J Biol Chem.

[30] Menendez-Benito V, Verhoef LG, Masucci MG, Dantuma NP (2005). Endoplasmic reticulum stress compromises the ubiquitin-proteasome system.. Hum Mol Genet.

[31] Yang M, Omura S, Bonifacino JS, Weissman AM (1998). Novel aspects of degradation of T cell receptor subunits from the endoplasmic reticulum (ER) in T cells: importance of oligosaccharide processing, ubiquitination, and proteasome-dependent removal from ER membranes.. J Exp Med.

[32] Hipp MS, Kalveram B, Raasi S, Groettrup M, Schmidtke G (2005). FAT10, a ubiquitin-independent signal for proteasomal degradation.. Mol Cell Biol.

[33] Wang XZ, Lawson B, Brewer JW, Zinszner H, Sanjay A (1996). Signals from the stressed endoplasmic reticulum induce C/EBP-homologous protein (CHOP/GADD153).. Mol Cell Biol.

[34] Bertolotti A, Zhang Y, Hendershot LM, Harding HP, Ron D (2000). Dynamic interaction of BiP and ER stress transducers in the unfolded-protein response.. Nat Cell Biol.

[35] Kozutsumi Y, Segal M, Normington K, Gething MJ, Sambrook J (1988). The presence of malfolded proteins in the endoplasmic reticulum signals the induction of glucose-regulated proteins.. Nature.

[36] Boyce M, Bryant KF, Jousse C, Long K, Harding HP (2005). A selective inhibitor of eIF2alpha dephosphorylation protects cells from ER stress.. Science.

[37] Watanabe M, Dykes-Hoberg M, Culotta VC, Price DL, Wong PC (2001). Histological evidence of protein aggregation in mutant SOD1 transgenic mice and in amyotrophic lateral sclerosis neural tissues.. Neurobiol Dis.

[38] Lev S, Ben Halevy D, Peretti D, Dahan N (2008). The VAP protein family: from cellular functions to motor neuron disease.. Trends Cell Biol.

[39] Fan JQ, Ishii S, Asano N, Suzuki Y (1999). Accelerated transport and maturation of lysosomal alpha-galactosidase A in Fabry lymphoblasts by an enzyme inhibitor.. Nat Med.

[40] Valentine JS, Doucette PA, Zittin Potter S (2005). Copper-zinc superoxide dismutase and amyotrophic lateral sclerosis.. Annu Rev Biochem.

[41] Diestel S, Schaefer D, Cremer H, Schmitz B (2007). NCAM is ubiquitylated, endocytosed and recycled in neurons.. J Cell Sci.

[42] Kabuta T, Suzuki Y, Wada K (2006). Degradation of amyotrophic lateral sclerosis-linked mutant Cu,Zn-superoxide dismutase proteins by macroautophagy and the proteasome.. J Biol Chem.

[43] Selkoe DJ (2001). Clearing the brain's amyloid cobwebs.. Neuron.

[44] Nalivaeva NN, Fisk LR, Belyaev ND, Turner AJ (2008). Amyloid-degrading enzymes as therapeutic targets in Alzheimer's disease.. Curr Alzheimer Res.

[45] Jung T, Grune T (2008). The proteasome and its role in the degradation of oxidized proteins.. IUBMB Life.

[46] Kaganovich D, Kopito R, Frydman J (2008). Misfolded proteins partition between two distinct quality control compartments.. Nature.

[47] Saxena S, Cabuy E, Caroni P (2009). A role for motoneuron subtype-selective ER stress in disease manifestations of FALS mice.. Nat Neurosci.

[48] Scheper W, Hoozemans JJ (2009). Endoplasmic reticulum protein quality control in neurodegenerative disease: the good, the bad and the therapy.. Curr Med Chem.

[49] Cummings CJ, Mancini MA, Antalffy B, DeFranco DB, Orr HT (1998). Chaperone suppression of aggregation and altered subcellular proteasome localization imply protein misfolding in SCA1.. Nat Genet.

[50] Rao SN, Maity R, Sharma J, Dey P, Shankar SK (2010). Sequestration of chaperones and proteasome into Lafora bodies and proteasomal dysfunction induced by Lafora disease-associated mutations of malin.. Hum Mol Genet.

[51] Mendonca DM, Chimelli L, Martinez AM (2006). Expression of ubiquitin and proteasome in motorneurons and astrocytes of spinal cords from patients with amyotrophic lateral sclerosis.. Neurosci Lett.

[52] Vembar SS, Brodsky JL (2008). One step at a time: endoplasmic reticulum-associated degradation.. Nat Rev Mol Cell Biol.

[53] Nishitoh H, Kadowaki H, Nagai A, Maruyama T, Yokota T (2008). ALS-linked mutant SOD1 induces ER stress- and ASK1-dependent motor neuron death by targeting Derlin-1.. Genes Dev.

[54] Fricke B, Heink S, Steffen J, Kloetzel PM, Kruger E (2007). The proteasome maturation protein POMP facilitates major steps of 20S proteasome formation at the endoplasmic reticulum.. EMBO Rep.

[55] Matias AC, Ramos PC, Dohmen RJ (2010). Chaperone-assisted assembly of the proteasome core particle.. Biochem Soc Trans.

[56] Schafer MK, Nam YC, Moumen A, Keglowich L, Bouche E (2010). L1 syndrome mutations impair neuronal L1 function at different levels by divergent mechanisms.. Neurobiol Dis.

[57] Raoul C, Abbas-Terki T, Bensadoun JC, Guillot S, Haase G (2005). Lentiviral-mediated silencing of SOD1 through RNA interference retards disease onset and progression in a mouse model of ALS.. Nat Med.

[58] Aebischer J, Cassina P, Otsmane B, Moumen A, Seilhean D (2011). IFNgamma triggers a LIGHT-dependent selective death of motoneurons contributing to the non-cell-autonomous effects of mutant SOD1.. Cell Death Differ.

